# How Does National Scientific Funding Support Emerging Interdisciplinary Research: A Comparison Study of Big Data Research in the US and China

**DOI:** 10.1371/journal.pone.0154509

**Published:** 2016-05-24

**Authors:** Ying Huang, Yi Zhang, Jan Youtie, Alan L. Porter, Xuefeng Wang

**Affiliations:** 1 School of Management and Economics, Beijing Institute of Technology, Beijing, 100081, China; 2 Centre for Quantum Computation and Intelligent Systems, Faculty of Engineering and Information Technology, University of Technology, Sydney, NSW 2007, Australia; 3 Enterprise Innovation Institute, Georgia Institute of Technology, Atlanta, GA, 30332, United States of America; 4 School of Public Policy, Georgia Institute of Technology, Atlanta, GA, 30332, United States of America; University of Sheffield, UNITED KINGDOM

## Abstract

How do funding agencies ramp-up their capabilities to support research in a rapidly emerging area? This paper addresses this question through a comparison of research proposals awarded by the US National Science Foundation (NSF) and the National Natural Science Foundation of China (NSFC) in the field of Big Data. Big data is characterized by its size and difficulties in capturing, curating, managing and processing it in reasonable periods of time. Although Big Data has its legacy in longstanding information technology research, the field grew very rapidly over a short period. We find that the extent of interdisciplinarity is a key aspect in how these funding agencies address the rise of Big Data. Our results show that both agencies have been able to marshal funding to support Big Data research in multiple areas, but the NSF relies to a greater extent on multi-program funding from different fields. We discuss how these interdisciplinary approaches reflect the research hot-spots and innovation pathways in these two countries.

## Introduction

Dramatic advances resulting from the rapid pace of technological developments and new interdisciplinary fields are on the horizon [1]. Science and engineering research continually evolves beyond the boundaries of single disciplines and offers employment opportunities that require not only depth of knowledge but also breadth of knowledge, integration, synthesis, and an array of skills. Interdisciplinary research (IDR), defined as work which integrates theories, methods, tools and/or concepts from multiple specialized knowledge bases, is often treated as proxy for research to advance fundamental understanding or to solve problems whose solutions are beyond the scope of a single discipline or area of research practice [2, 3]. Since IDR is increasingly prominent today, compiling reflections on its premises and issues could serve those engaged in R&D analysis, policy and management [4], and accelerate the process of scientific discoveries and societal problem solving.

Interdisciplinarity has three distinct components that are typically taken into account: Variety, Balance and Disparity [5]. Shannon entropy [6] and Simpson [7] offered popular diversity indices in the ecological literature and more recently in the interdisciplinarity literature to capture both variety and balance of referenced disciplines. Furthermore, Rao [8] and Stirling [5] proposed disparity as the third dimension, and Rao-Stirling diversity was a composite measure of the three diversity components [5]. Further indicators, based on the above theoretical foundations, have been proposed to measure IDR, including network coherence [9], specialization scores [10], the integration score [11, 12], the diffusion score [13], and other improved indicators [14, 15]. However, most of bibliometric literatures on measuring IDR focuses on the outputs of science, or publications [16]. Less emphasis has been placed on inputs, such as research proposals, which typically contain broader information than research articles. Proposals reflect on academic individuals, networks, and evolutionary processes of science and directly indicate overarching research programs, which, if funded, can lead to multiple publications [17]. In general, research proposals are granted by national governments and aimed to support academic institutions and R&D departments to conduct basic research, the content of which focuses on new ideas, concepts, and potential innovative actions. Understanding of funding proposals could be considered as an express path to reveal how evolutionary R&D pathways work in given countries, regions, and research fields [18].

Scientific funding plays an essential role in individual scientific research, university discipline construction, and national innovation system patterns [19]. Previous research on scientific funding has focused on research investments [20], optimization design for peer review [21], and funding distribution [22]. In addition, evaluations of the social impact [23, 24] and performance [25, 26] of science funding reflect an evaluative perspective on funding research.

As science increasingly deals with boundary-spanning problems, various policy and funding initiatives have been developed to encourage interdisciplinary research to push forward academic capability and accelerate scientific discovery. National scientific funding organizations specifically support research, including investigations of an interdisciplinary nature, through a variety of target methods [24]. As the two leading scientific funding organizations in the world, National Science Foundation (NSF) of the US and National Natural Science Foundation of China (NSFC) are noteworthy. NSFC, as the largest scientific funding agency in China, has made considerable efforts to promote basic and applied research [27]. NSFC not only supports cutting-edge ideas and projects in different disciplines, but also sponsors high-level strategic academic exchange platforms (e.g. Shuangqing Forum) to promote interdisciplinary studies and innovative cultures and create a favorable environment for research [28]. Dr. Wei Yang, the present president of NSFC, defined strengthening interdisciplinary interaction as one key mission of NSFC [29]. The US NSF supports research and education in non-medical fields of science and engineering, not only to produce leading edge research, but also to narrow the gap between science and society through the use of broader impacts criteria [30, 31]. NSF treats fostering IDR as its high priority and encourages researchers to conduct IDR research in novel and emerging areas extending beyond any particular current NSF programs to keep the US at the leading edge of discovery in a wide range of scientific areas [32].

We are especially interested in examining how these two agencies support research in a rapidly growing interdisciplinary domain. The domain of interest for this study is Big Data. In an increasingly complex economic and social environment, accessing vast amounts of data and information can help organizations and governments make better policies, predictions and decisions [33]. Big Data is the application of data technology to obtain valuable information from various types of extremely large data sets, which can come from social networks, images, sensors, the web, or other unstructured sources [34]. Big Data has its legacy in information technology developments. However, its capacity and analytic capabilities promise to make an essential contribution in areas such as traffic management, logistics, health care, and education [35]. Big Data research can help further these scientific and societal benefits for governments, enterprises and academic institutions.

Aiming to make the most of the fast-growing volume of digital data, the US government established a “Big Data Research and Development Initiative” program on March 29, 2012, which committed over $200 million in new funding through six agencies to extract knowledge and insights from large and complex collections of digital data [36]. The Chinese government has similarly targeted Big Data as a strategic area of research and development [37]. Since 2012, a large number of Big Data-related projects have been supported by the Ministry of Science and Technology, the National Development and Reform Commission, the Ministry of Industry and Information Technology, and other central governmental departments of China. Porter and his colleagues [38] figured out that Big Data scientific publications grew dramatically in 2013 and 2014, by more than four times the number of the papers published in 2012. They further reported that the leading countries based on author location were the US and China; these two countries accounted for more than half of all Big Data publications and nearly all of the top 30 author organizations.

In this paper, we apply tech mining [i.e., text mining of science, technology & innovation (ST&I) information to generate useful intelligence] [39], network analysis, and interdisciplinary assessment methods to understand how NSF and NSFC have ramped-up their funding awards to address the rapid rise of Big Data research. Our particular focus is on research awards rather than scientific articles [40–43]. Our research addresses the following questions about how NSF and NSFC support research in rapidly emerging areas, based on a case study of the emergence of Big Data research:

1. How do NSF and NSFC differ in terms of the amount of funding resources allocated to Big Data research over time?
2. To what extent do NSF and NSFC fund similar or different subareas of Big Data research?
3. What are the disciplinary ranges and connections in Big Data funded proposals in the two organizations and how do they differ in the degree of interdisciplinarity of funding research?

The remainder of this paper consists of four sections. Following this general introduction, the “Methodology” section describes the framework and methodology. The “Results” section presents comparative results of the research profiling and tech mining analyses. The “Conclusion and Discussion” section reviews our research, identifies research limitations, and indicates promising research opportunities to pursue in the future.

## Methodology

Based on the aforementioned research questions, we retrieved a raw dataset of all funded research proposals relating to Big Data from the NSF official website (http://www.nsf.gov/) and the NSFC’s ISIS system (Internet-based Science Information System) (http://isisn.nsfc.gov.cn/). The NSF website offers two different search types: (1) simple search: provides title, abstract, names, institutions, programs and other information associated with an award; and (2) consolidated advanced search: provides access to all fielded searches. In this paper, we chose the consolidated advanced search.

The ISIS system was applied nationwide in 2003, and it has been well received and supports NSFC to implement managerial control to cope with China’s rapidly growing research productivity [44]. In the ISIS system, users can retrieve the information by typing specific search terms in the query page, but only a few fields can be viewed: Awards Number, Disciplinary Application Code, Title, Principal Investigator, Affiliation, Sponsored Funds, Start Date and End Date.

A key question is when to begin the search. First, we originally set the starting year at 2008, which is when a special issue of *Nature*, “what Big Data sets mean for contemporary science” [45], was published. However, there are no awards for the year 2008 in our retrieval of funded proposals. Thus, 2009 became our starting year. Second, we originally planned to use a topical search, which captures the occurrences of “Big Data” in titles, abstracts and keywords [46]. This approach worked for searching the NSF awards database. Ultimately, we obtained 921 records from NSF and 434 records from NSFC during the period of 2009 to 2015. However, because the NSFC data are in Chinese, we found that use of standard codes such as the NSFC’s Disciplinary Application Code (DAC), was more effective than direct translation of topics during the analyzing process. We validated the DACs further by arranging native speakers to examine our approach and select NSFC funded proposal records to guarantee its comparability.

We conducted a retrieval of publications from Web of Science (http://www.webofknowledge.com/) to verify some conclusions and judgments about searching the funded proposal databases using an elementary search strategy of TS = “Big Data”. This approach resulted in 2815 records during the period of 2008 to 2015 (retrieved on January 8, 2016). We imported the data into the text-mining tool VantagePoint (https://www.thevantagepoint.com/), and ascertained from the funding acknowledgements section of these papers that 272 publications were supported by NSFC, and 198 by NSF. They ranked as the top 2 national funding agencies, and occupied 22.48% and 16.36% respectively in all funded publications (1210 publications). All raw data of Big Data proposals funded by NSF and NSFC can be founded in “S1 File”.

In addition to seeking to measure basic activity information, we also attempted to interpret the reasons and rules behind the activities of these two funding agencies. To this end we drew on the work of tech mining research [47, 48] to extract metadata for research profiling. We also introduced two indicators to measure the inequality of funding organization distribution and the degree of interdisciplinarity for a single granted project.

**Fig 1:**
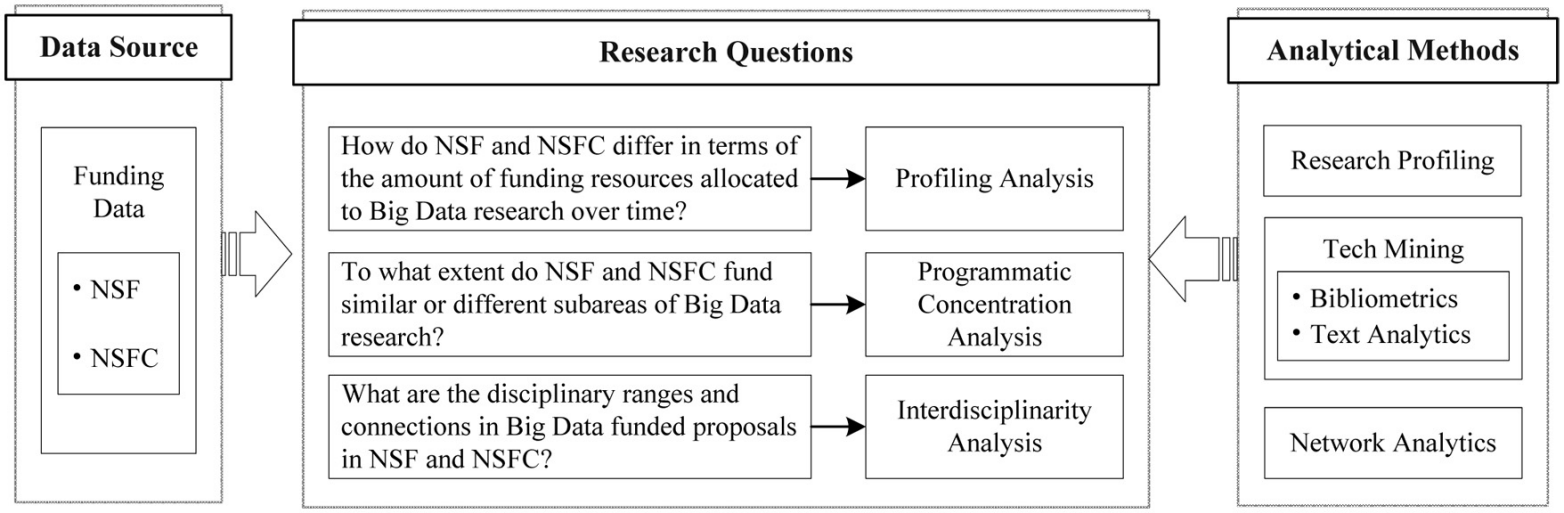
The Framework of Paper.

The framework of this paper, comprising the data resources, research questions, and analytical methods, is diagramed below (Fig 1).

## Results

### Research Profling Analysis

**Fig 2:**
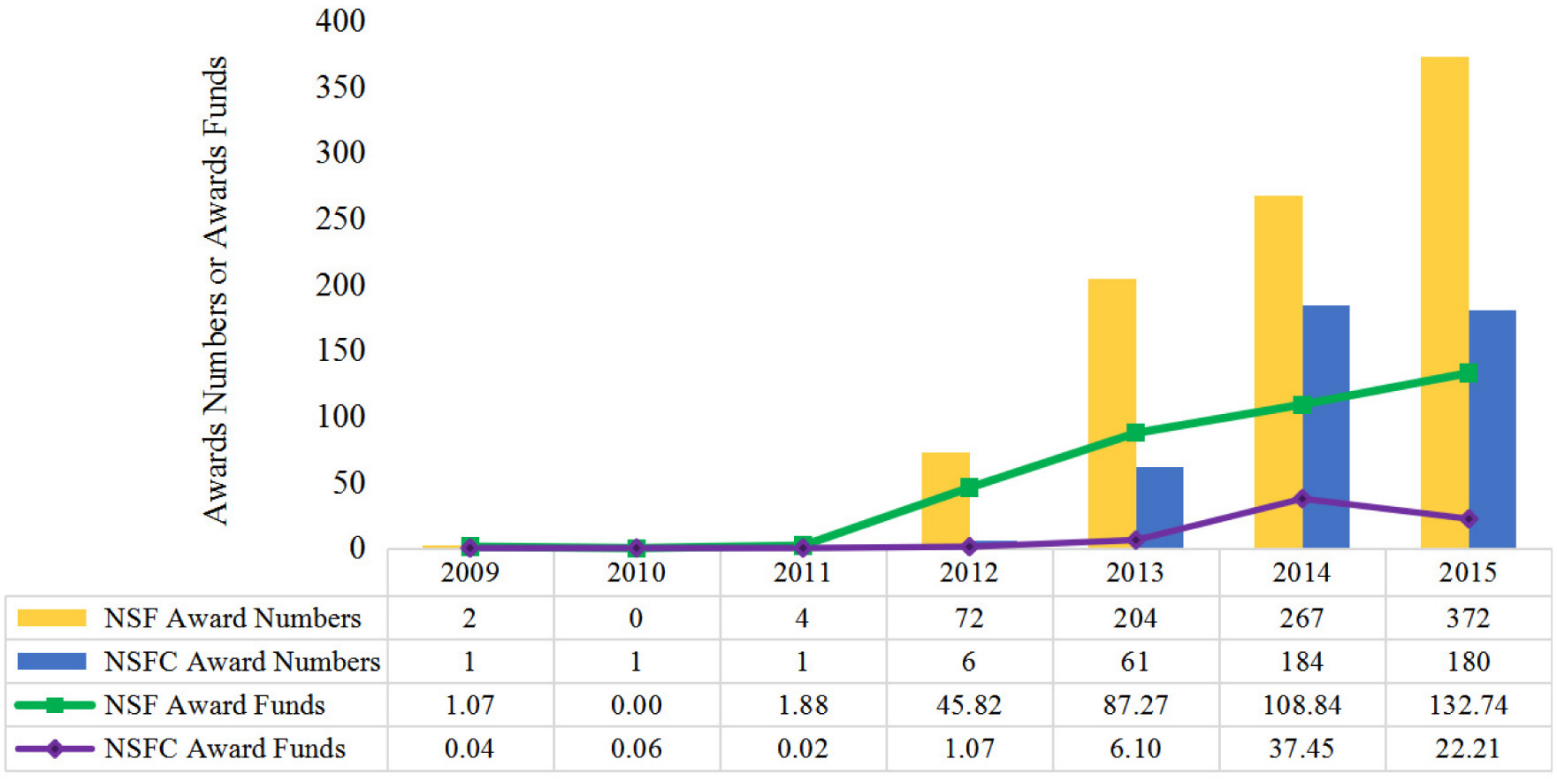
Summary of Big Data Awards Supported by NSF and NSFC. Note: NSFC funds data are transformed by using the exchange rates in current year.

Fig 2 shows grant activity trends for Big Data supported by NSF and NSFC from 2009 to 2015. Looking at the counts of grant awards, both NSF and NSFC supported very limited Big Data research before 2012. For NSF, we can see an increase in 2012; a notable rise in both counts of Big Data awards and dollar amounts associated with these counts can be observed. For NSFC, the number of granted projects increased from only 1 record in 2009 to 61 records in 2013; these figures for NSF are 2 and 204. In regards to funds granted, NSF sponsored universities and other organizations with $45.82 million in 2012, 42 times more than NSFC did ($1.07 million dollars). NSF and NSFC both continued to increase funding, reaching $108.84 million and $37.45 million in 2014, respectively. There is a slight downturn in NSFC award activity in 2015 which is not presented in the NSF award data. This downturn in NSFC award activity may not necessarily represent a decline in funding Big Data research in China. In China, nearly every province has its own science foundation (including Natural Science Foundation and Social Science Foundation). At the same time, several central departments also offer funding to support scientific research, such as the Ministry of Science and Technology of China, the Ministry of Education of China, and so on. While a parallel system exists for the US (through research funding by state governments, other federal agencies, and private non-profit organizations), the US system is not as extensive as is the case in China.

Why was 2013 such a bellwether year for Big Data research proposals in both countries? We are not privy to all the reasons for this growth, but one factor is likely to be the White House’s announcing the “Big Data Research and Development Initiative” in 2012. NSF subsequently announced its support of new research to extract knowledge and insights from large and complex collections of digital data, including developing new methods of deriving knowledge from data; constructing a new infrastructure to manage, curate and deliver data to communities; and forging new approaches for associated education and training [49]. Four specific programs were set up through the NSF’s Computer and Information Science and Engineering Directorate (CISE). One of the most important programs is the Critical Techniques and Technologies for Advancing Foundations and Applications of Big Data Science & Engineering (BIGDATA Program). Multiple NSF directorates and other federal agencies participated in this program. The research directions of NSFC are influenced by NSF to some extent. At the same time, NSFC began to organize the high-level Big Data-related “Shuangqing Forum” academic workshop in 2013 to focus on national strategic development. This workshop was repeated in subsequent years, which suggested NSFC’s persistent value towards the Big Data area for strategic research investment (Table 1). These forums proposed several frontiers for crucial scientific problems and suggested policies and solutions associated with research on technologies and applications of Big Data [50]. Moreover, the topical focus progressed from an emphasis on problems in the Shanghai workshop in 2013 to methods two years later at the Guangzhou workshop.

**Table 1:** The Profile of Big Data- Related Shuangqing Forum.

Workshop ID	Topic	Time	Place	Organizers
**89th**	Challenging scientific problems in the fields of technologies and applications of Big Data	March 5–7, 2013	Shanghai	NSFC, Tongji University
**109th**	Big data and major basic problems on management and decisions	March 26–27, 2014	Beijing	NSFC
**132th**	Multi-disciplinary methods for Big Data analysis and processing	March 26–27, 2015	Guangzhou	NSFC, Sun Yat-sen University

What type of projects resulted from these research investments? Table 2 indicates the proportion of granted projects by organization type in the US and China. Both NSF and NSFC tended to emphasize academic research, which accounted for 92.83% and 88.25% of the total number of funded proposals. This finding is not surprising given NSF and NSFC are the agencies with an orientation toward providing support for academic research [51]. At the same time, NSFC awards are less intensely focused on universities than are NSF’s. This lesser focus lies in the importance of research institutes in the Chinese research and innovation system, particularly the Chinese Academy of Sciences. The Chinese Academy of Sciences is seen as the linchpin of China’s drive to explore and harness high technology and the natural sciences for the benefit of China. Thus, it is not surprising that the Chinese Academy of Science would be prominent among institutions receiving awards from NSFC. About 11.29% of NSFC’ grants were granted to research institutes compared to only 2.28% for NSF. However, NSFC does not support research funding by individuals and corporations, so none of NSFC’s funding went to the private sector. Nearly 4.23% of awards were conferred on individuals and corporations by NSF through grants, and cooperative agreements.

**Table 2:** Grant Recipient Organizations: NSF and NSFC.

Organization Type	Numbers of NSF Awards	% of Total NSF Big Data Awards	Numbers of NSFC Awards	% of Total NSFC Big Data Awards
Academic University	855	92.83	383	88.25
Research Institution	21	2.28	49	11.29
Corporation	39	4.23	0	0.00
Other	6	0.65	2	0.46

Note: *Academic University* is the organization granting academic degrees in various subjects; *Research Institution* is an independent, nonprofit research institute; *Corporation* is a company or group of people authorized to purse profit-making rather than non-profit; *Other* includes individuals, hospitals and other research organizations.

We provided a further measure of the extent of concentration of the distribution of funding by NSF and NSFC. In this context, we applied a normalized Gini coefficient to measure the inequality of funding organization distribution, both in project counts and funding amounts. The Gini index was originally proposed to measure income inequality, but it also has been used to capture the inequality, unevenness, and imbalance of the distribution of references across involved disciplines. Higher Gini coefficients represent greater inequality, in this case, of scientific funding distribution across organizations. The normalized Gini coefficient can be calculated as [52]:

$$G_{n}=\frac{{\sum^{\;}}_{i=1}^{n}\left(2i-n-1\right)X_{i}}{\left(n-1\right){\sum^{\;}}_{i=1}^{n}X_{i}}$$ (1)

In the eq (1), *n* indicates the number of sponsored organizations and *n>1*, *i* is the ranking of *n*, and *x*_*i*_ presents the number of projects or the amount of funds obtained by the *i* ^*th*^ organization.

**Fig 3:**
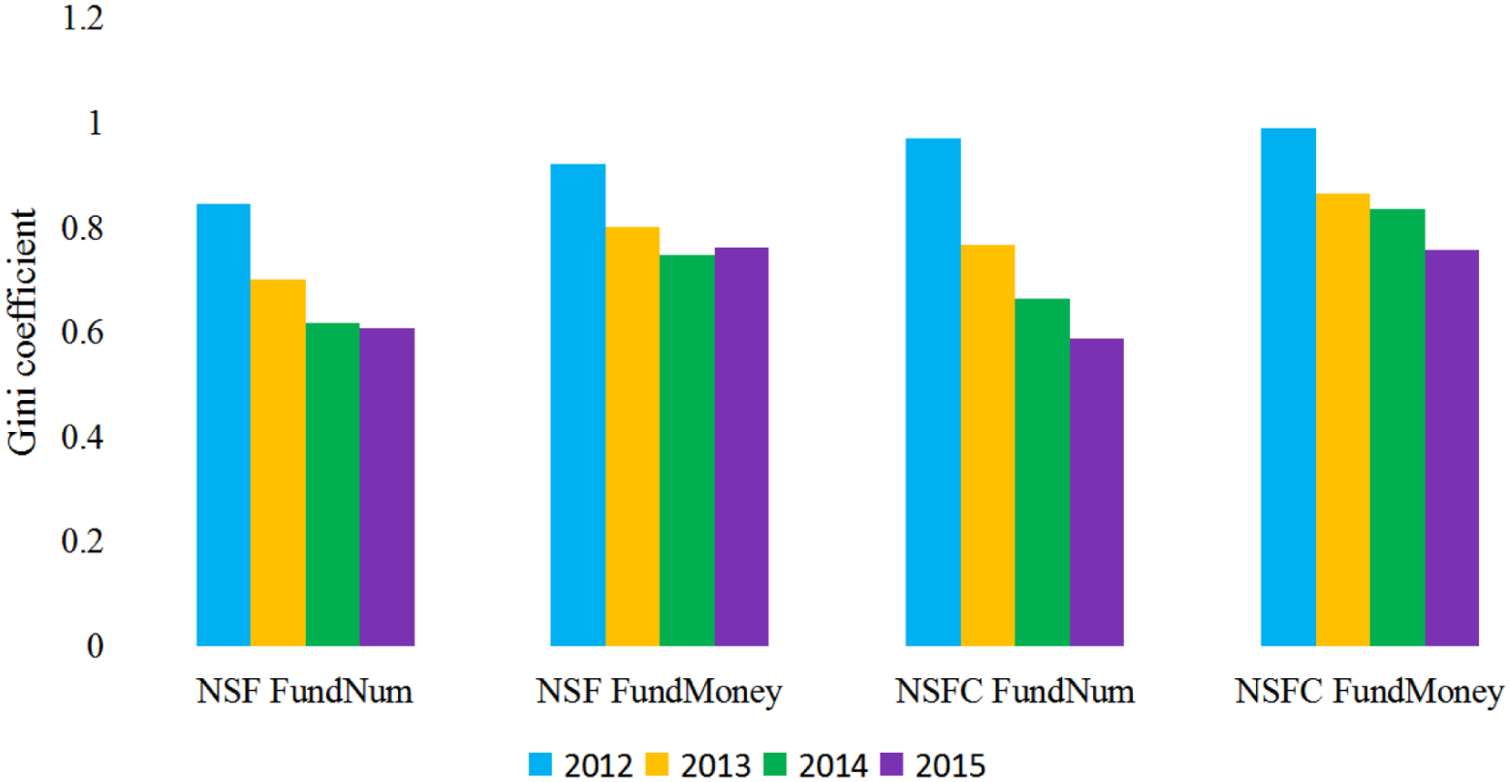
Gini Coefficient in Terms of the Number of Grants (FundNum) the Amount of Sponsored Money (FundMoney) of NSF and NSFC.

We applied this measure to the distribution of organizations receiving grants from the two agencies over the 2012-to-2015 period because the time after 2012 comprised the bulk of the grants. The results indicated different distributions for the number of grants (FundNum) and the amount of sponsored money (FundMoney) (Fig 3). In terms of the FundNum, NSF has a relatively lower Gini coefficient than does NSFC. NSFC’s Gini coefficient was much lower in 2015 than in 2012. Furthermore, when one looks at the distribution of money, we can see that NSFC’s Gini coefficient was slightly higher than NSF’s in 2012, but coefficients associated with the two sponsors were relatively similar by 2015. NSF’s coefficient associated with the distribution of money exhibited a slight decrease over the three years while NSFC’s coefficient was decreasing more sharply.

Based on the Gini coefficient analysis, we can see that the Gini coefficients were very high in the early stages, in terms of granted numbers and funds, at both sponsoring agencies. It is inferred that in the beginning stage of emerging interdisciplinary research, relatively few organizations care about, or have considerable influence on, such newly-formed topics. From the overall trends of the Gini coefficient, the awards distribution became broader with the advance of Big Data studies, and the number of grants was more balanced between different organizations than the amount of sponsored money bestowed.

### Proposal Programmatic Concentration Analysis

The extent to which funding is concentrated or spread among multiple disciplinary programs in a funding agency may well be an important factor in understanding the development of research in a rapidly emerging field. The NSF is organized into directorates that align with broad scientific disciplines: Biological Sciences (BIO), Computer & Information Science & Engineering (CISE), Education & Human Resources (EHR), Engineering (ENG), Geosciences (GEO), Mathematical & Physical Sciences (MPS), Social, Behavioral & Economic Sciences (SBE) and Other (non-disciplinary specific). Because MPS houses several markedly disparate disciplines, the MPS divisions (Astronomical Sciences, Chemistry, Material Sciences, Mathematics, and Physics) are often used instead [17]. In this paper, we treat the divisions under these directorates as different research areas and consider the NSF’s detailed programs as a proxy for research fields. Similarly, in the NSFC, there are eight scientific departments: Mathematical and Physical Science (A), Chemical Sciences (B), Life Sciences (C), Earth Sciences (D), Engineering and Materials Sciences (E), Information Sciences (F), Management Sciences (G) and Medical Sciences (H) (The department of Medical Sciences in NSFC established in 2010). For each project, NSFC applicants must provide a DAC in order to select suitable peer reviewers and help classify the project in its evaluation. DAC is a three-level code indicating the detailed discipline to which an application belongs; it is composed of English characters and Arabic numerals. The English character is the code of a scientific department. The three levels of Arabic numerals denote research areas, research fields and research directions, respectively. The detailed research areas and their corresponding codes are shown in S1 Table.

Although NSF and NSFC have different disciplinary categorizations, most of these categories can be matched. Table 3 indicates that the categories of Information Sciences and Computer & Information Science & Engineering account for the largest number of proposed projects. Big Data has conventionally been considered as a part of information sciences since it is the process of mining potential information from voluminous amounts of structured, semi-structured and/or unstructured data. Big Data also has a very close relationship with information and computer technologies, including data collection, storage, processing, and analysis/visualization [53]. For NSF, the second ranking discipline is Engineering, but for NSFC, it is Management Sciences. This shows that US researchers cared more about practical applications in specialized engineering fields while Chinese scholars are more interested in strategic planning to improve decision-making in critical development areas, such as healthcare, social administration, environment protection and resource management. One factor underlying this difference is that China and US are at different stages of development, so Big Data is sometimes treated as a powerful tool to solve practical issues in the US but as a tool for management reform in China.

**Table 3:** Disciplinary Composition of the Big Data Awards in NSF and NSFC.

NSF Discipline	Records	% of Total NSF Big Data Awards	NSFC Discipline	Records	%of Total NSFC Big Data Awards
Computer & Information Science & Engineering (CISE)	511	55.48	Information Sciences (IS)	236	54.38
Engineering (ENG)	134	14.55	Management Sciences (MaS)	111	25.58
Mathematical & Physical Sciences (MPS)	127	13.79	Engineering & Materials Sciences (EMS)	30	6.91
Social, Behavioral & Economic Sciences (SBE)	61	6.62	Earth Sciences (ES)	18	4.15
Education & Human Resources (EHR)	38	4.13	Mathematical & Physical Sciences (MPS)	17	3.92
Biological Sciences (BIO)	34	3.69	Medical Sciences (MeS)	10	2.30
Geosciences (GEO)	12	1.30	Chemistry Sciences (CS)	2	0.46
Other	4	0.43	Life Sciences (LS)	1	0.23
			Other	9	2.07

In order to obtain insights into the specific research areas and their main research fields (more than 15 projects belong to a certain area) between Computer & Information Science & Engineering (CISE) of NSF and Information Sciences (IS) of NSFC, we organized them in Table 4 (for NSF) and Table 5 (for NSFC). For NSF, the main four research areas in CISE were Information & Intelligent Systems (189 records, 20.52%), Computing & Communication Foundations (114 records, 12.38%). Computer & Network Systems (107 records, 11.62%) and Advanced Cyberinfrastructure (101 records, 10.97%) and. The top research fields within these areas were Big Data Science & Engineering, Info Integration & Informatics, Campus Cyberinfrastructure and Computer Systems.

**Table 4:** Research Areas and Main Research Fields of CISE in NSF.

Research Areas and Main Research Fields	Records	% of Total NSF Big Data Awards
Information & Intelligent Systems	189	20.52
*Big Data Science & Engineering	60	-
*Info Integration & Informatics	51	-
*Information Technology Research	17	-
Computing & Communication Foundations	114	12.38
*Communication & Information Foundations	26	-
*Algorithmic Foundations	21	-
*Software & Hardware Foundation	18	-
Computer & Network Systems	107	11.62
*Computer Systems	31	-
*Computing research infrastructure	10	-
Advanced Cyberinfrastructure	101	10.97
*Campus Cyberinfrastructure	37	-

* indicates the research fields.

**Table 5:** Research Areas and Main Research Fields of IS in NSFC.

Research Areas and Main Research Fields	Records	% of Total NSFC Big Data Awards
Computer Science	170	39.17
*Computer Applications Technology	65	-
*Computer Software	41	-
*Computer Network	23	-
*Computer Architecture	17	-
Automation	47	10.83
*Artificial Intelligence & Knowledge Engineering	18	-
Electronics & Information System	19	4.38

* indicates the research fields.

Although we could not create a side-by-side comparison on research fields between NSF and NSFC, it was still possible to compare and contrast the top funding fields and hot-spots in Tables 4 and 5. As the comparison in topics relies heavily on the accuracy of the translations, we attempted to conduct this analysis for NSFC based on the DAC that included subject classification information, and then translated the main research fields corresponding to the DAC. The results indicate that NSFC primarily funds Big Data research out of the Computer Science area, which comprises nearly 39.17% of all Big Data awards. Another 10.83% of awards are conferred by the Automation area and 4.38% by the Electronics & Information System. Within the Computer Science area, NSFC has balanced the number of awards across several different scientific directions. Computer Applications Technology, Computer Software, Computer Network and Computer Architecture are important fields in the Computing Science research area. Additionally, other prominent research field is Artificial Intelligence & Knowledge Engineering (18 records) in the Automation area (47 records).

When proposers apply for funding from the NSFC, they are required to provide project terms or keywords. As far as we know, keywords are not required for NSF proposals and are therefore not comparably available on the NSF website. To address the lack of investigator-provided keywords, we conducted Natural Language Processing (NLP) on the proposal title field. We used the title field, rather than the abstract field, because the terms in the title were more distinctive. We extracted phrases from the title by applying NLP with the support of the text-mining tool suite- VantagePoint. Phrases and terms retrieved in this way are large and "noisy," making them difficult to manually categorize. Using bibliometric and text mining techniques, this paper applied semi-automated "Term Clumping" to generate better term lists for achieving competitive technical intelligence [54]. For the NSFC awards information, we first extracted the title terms and uploaded them to the LTP-Cloud (Language Technology Platform Cloud) (http://www.ltp-cloud.com/) to process Chinese word segmentation. After obtaining a list of phrases, we imported these keywords into our own Chinese text analysis tools—ItgInsight—to help us conduct text cleaning. This process was comprised of four steps: (1) Common and basic term removal, e.g., instance, technology; (2) Fuzzy word matching (to combine terms with similar structures based on pattern commonality, such as stemming—e.g., institute and institution, and combining singular and plural forms of English words, e.g., technology and technologies); (3) Extreme word removal [to remove very common (top 5%) and very rare (occurrence only in single records) terms]; (4) Combine term networks (to combine selected low-frequency phrases with the high-frequency phrases that appear in the same records, and sharing terms). We then translated the top 50 high-frequency Chinese phrases and invited some postgraduates with English language background or bachelor’s degree to validate the translation.

**Fig 4:**
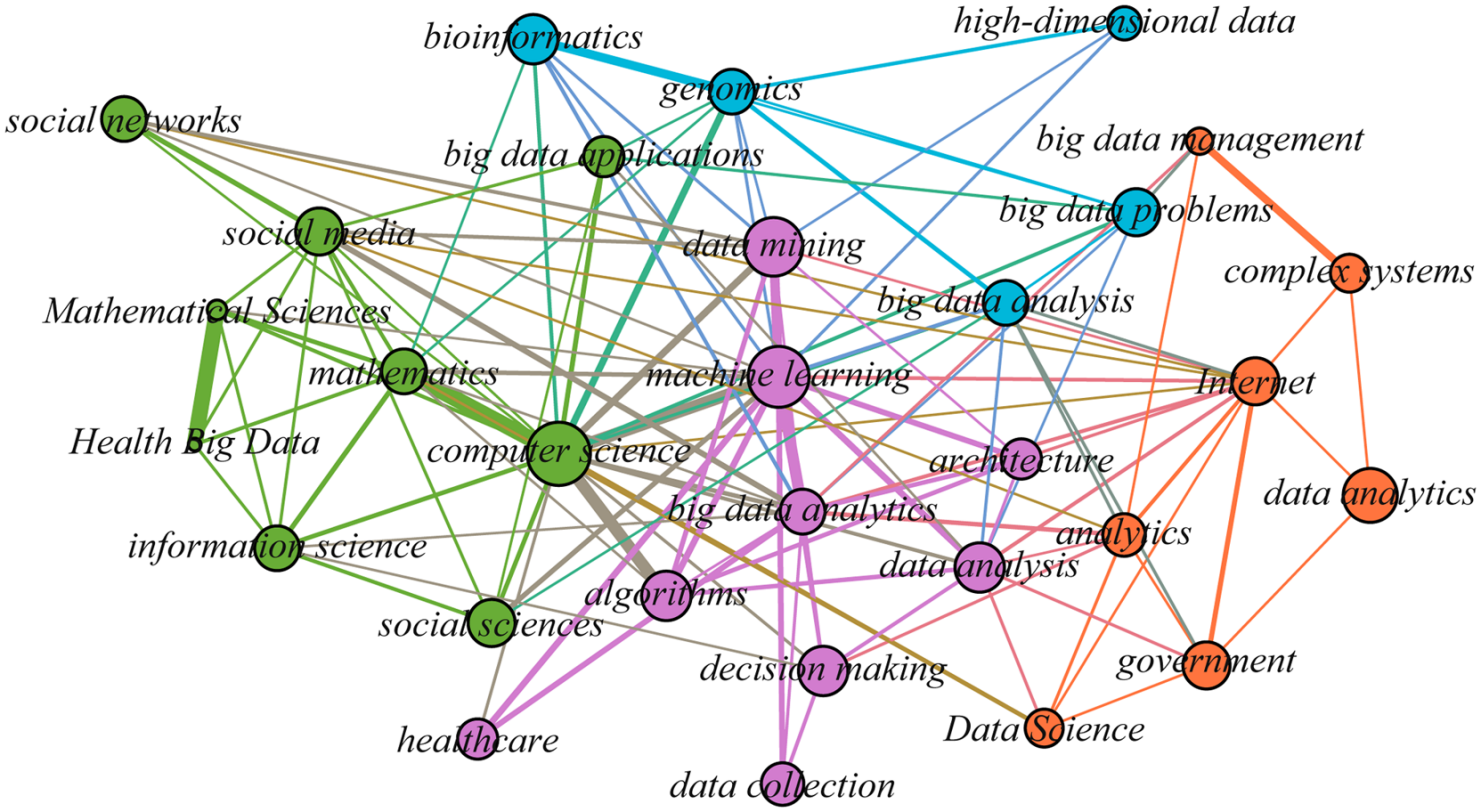
Semantic Network of 30 Most Frequently Occurring Terms in NSF Big Data Proposals.

**Fig 5:**
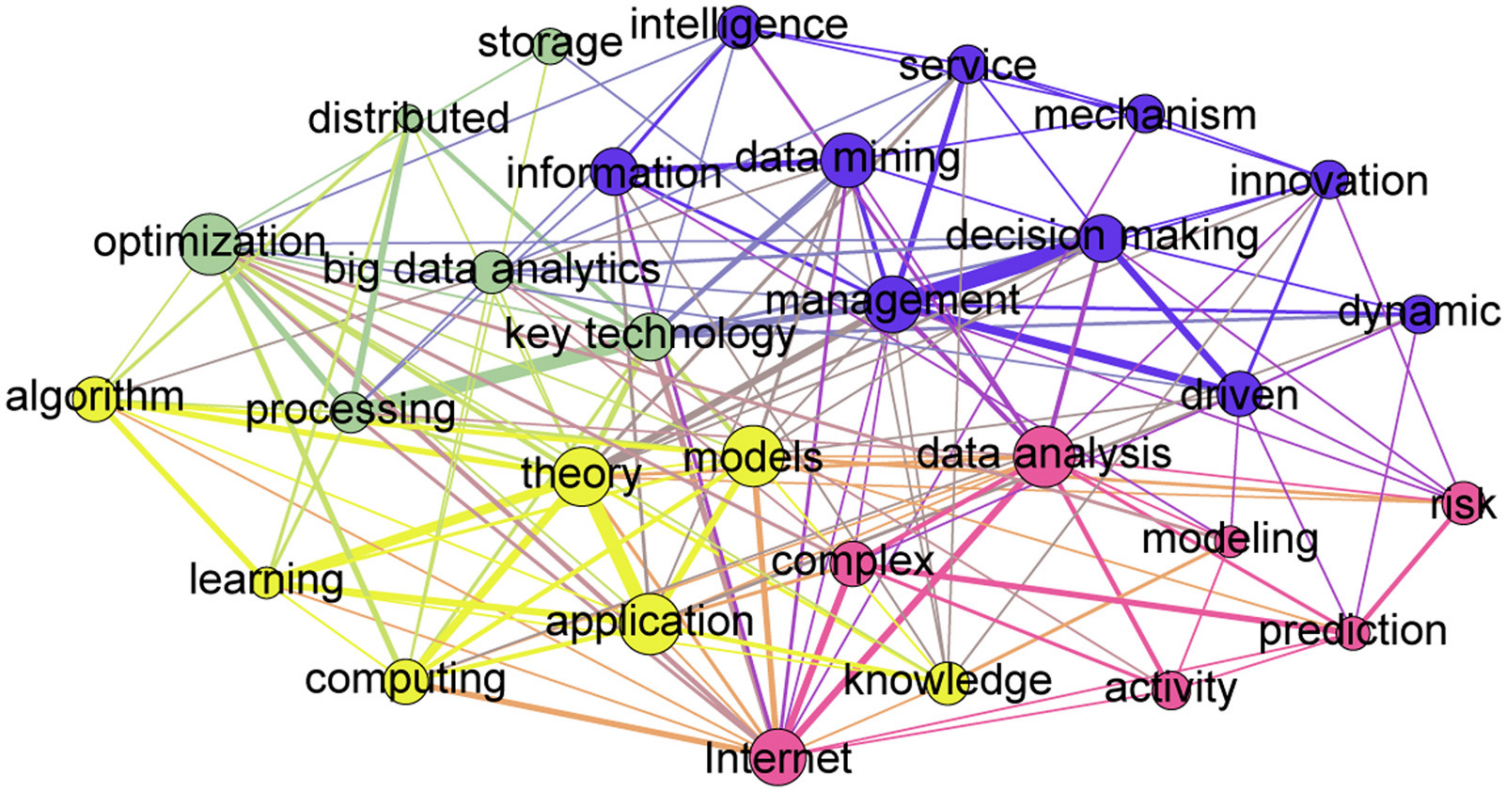
Semantic Network of 30 Most Frequently Occurring Terms in NSFC Big Data Proposals.

Before visualizing the semantic networks based on these keywords, we calculated the frequency of specific terms. The semantic networks of the 30 most frequently occurring terms in Big Data are shown in Fig 4 for NSF and Fig 5 for NSFC, which are mapped in the visualization and exploration software- Gephi (http://gephi.github.io/). The node size represents the frequency of a term in proposal titles, and the linkages among different terms are based on their co-occurrence in proposal titles. The color of the node is used to distinguish whether these nodes belong to the same cluster or not, and the cluster membership was obtained from the community detection algorithm [55].

The 30 most frequently occurring terms in NSF granted proposals were clustered into four groups as follows (Fig 4):

- Green group represents the use of social network analysis in Big Data area; some related branch subjects and keywords are “social networks,” “social sciences,” “social media,” “mathematical sciences,” “mathematics,” “computer science,” “big data applications”; “Health Big Data” and “information science”;
- Purple group indicates some Big Data analytics techniques; keywords include: “data mining,” “machine learning,” “architecture,” “big data analytics,” “data analysis,” “algorithms,” “decision making,” “healthcare” and “data collection”;
- Blue group presents the problems and potential solutions in bioinformatics; key terms include: “high-dimensional data,” “bioinformatics,” “genomics,” “big data problems” and “big data analysis”;
- Orange group displays public management factors in the context of Big Data through keywords such as “big data management,” “complex systems,” “Internet,” “data analytics,” “analytics,” “government” and “data science”.

Following the above analytic logic, the 30 most frequently occurring terms in NSFC granted proposals were also clustered into four clusters (Fig 5):

- Orange cluster indicates the main theory and techniques in Big Data fields; the main phrases in this cluster are: “algorithm,” “machine learning,” “computing,” “models,” “theory,” “knowledge” and “application”;
- Blue cluster layouts highlight technologies involved with Big Data analysis; related phrases include: “optimization,” “processing,” “big data analytics,” “key technology,” “storage” and “distributed”;
- Green cluster presents the role of Big Data in complex environments through terms such as “Internet,” “complex,” “activity,” “data analysis,” “modeling,” “prediction” and “risk”;
- Purple cluster illustrates the use of Big in management practice, through key terms such as “information,” “data mining,” “management,” “intelligence,” “driven,” “decision making,” “knowledge,” “service,” “dynamic,” “mechanism” and “innovation”.

These results indicate that both NSF’s and NSFC’s proposals have considerable common research interests and foci on topics such as “algorithms,” “Internet,” “data analysis,” “data mining,” “decision making” and “machine learning. At the same time, there are distinctions in that the US concentrates more on engineering and specialized applications while China shows more emphasis on Big Data theory and concepts in management-related domains.

Based on the above proposal concentration analysis, we can see that the two countries share similar research topics for building up the Big Data field. However, since the two countries’ strategic goals and scientific development models may differ, the detailed concepts, techniques, and applications to which funding agencies pay attention are somewhat different.

### Proposal Interdisciplinarity Analysis

IDR has become a significant thrust of NSF and other scientific agencies in recent years. Most bibliometric literatures on measuring IDR focus on examining the outputs of science—publications [16]. Following this traditional evolution, we extracted the grant numbers from NSF’s and NSFC’s Big Data awards and retrieved the papers acknowledging them from the Web of Science. The results show that 428 publications were supported by NSF Big Data awards and 644 publications by NSFC (Retrieved on Jan 3, 2016). As an initial way to understand the disciplinary orientation of these publications, we analyzed the Web of Science Categories (“WCs”), which are assigned to journals based on a combination of cross-citation patterns and editorial judgment. WCs offer a standard in bibliometric analysis for representing disciplinary or field participation [4]. Table 6 shows the Top 10 WCs for Big Data publications supported by NSF and NSFC. Not surprisingly, most of the funded papers are in the Computer Science and Engineering, Electrical & Electronic areas. Multidisciplinary sciences and interdisciplinary studies are other foci in the funded papers. NSFC funded papers also pay attention to some management issues such as Operations Research & Management Science.

**Table 6:** Top 10 WCs for Big Data Publications Supported by NSF and NSF.

WCs	Records Supported by NSF	WCs	Records Supported by NSFC
Engineering, Electrical & Electronic	118	Computer Science, Artificial Intelligence	174
Computer Science, Information Systems	65	Engineering, Electrical & Electronic	147
Statistics & Probability	50	Computer Science, Information Systems	130
Computer Science, Software Engineering	49	Telecommunications	71
Computer Science, Artificial Intelligence	46	Computer Science, Software Engineering	53
Computer Science, Hardware & Architecture	34	Computer Science, Interdisciplinary Applications	51
Computer Science, Theory & Methods	34	Computer Science, Theory & Methods	48
Telecommunications	31	Engineering, Multidisciplinary	41
Multidisciplinary Sciences	26	Mathematics, Interdisciplinary Applications	39
Mathematical & Computational Biology	25	Operations Research & Management Science	39

**Fig 6:**
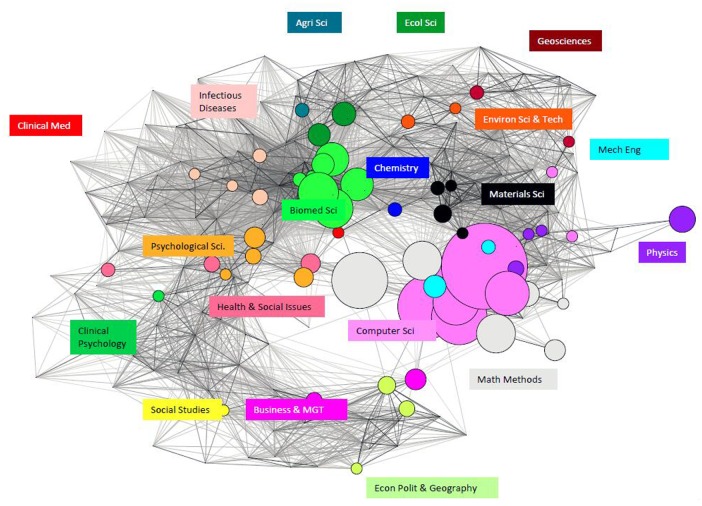
Big Data Research across the Disciplines of Publications Supported by NSF.

**Fig 7:**
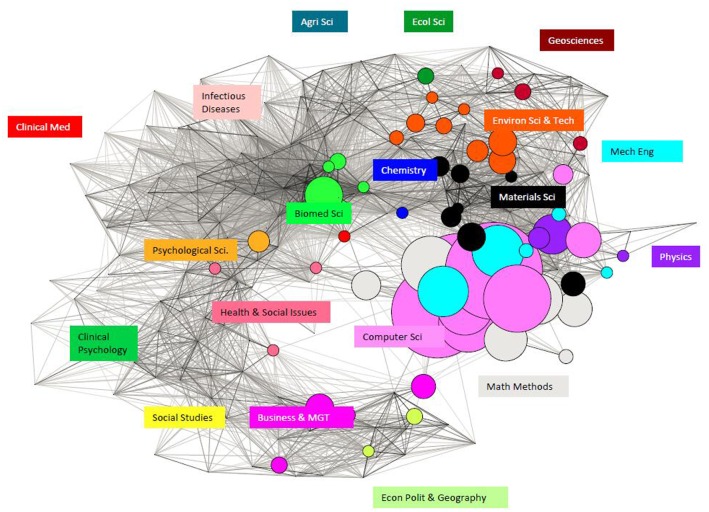
Big Data Research across the Disciplines of Publications Supported by NSFC.

We are particularly interested in cross-field research knowledge transfer and overall discipline distribution. For such purposes the granularity of the WCs is effective—i.e., some 224 WCs differentiate sub-fields, so we have applied science overlay mapping to visualize these differences [56] in Fig 6. and Fig 7. Again, not surprisingly, Big Data papers from NSF or NSFC funding are dominated by Computer Science, followed by Math Methods. But the pattern of widespread engagement is remarkable, suggesting that Big Data research is not bottled up in a silo [38]. There are plenty of Big Data papers from sponsored research related to Biomedicine Science in the case of NSF and Environment Science & Technology in the case of NSFC.

Next, we examine interdisciplinarity from the view of funding information. For NSFC applications, each proposer is required to provide a main DAC and a secondary DAC. The main DAC is used to select peer reviewers, while the second DAC is used to provide alternatives in case the main DAC creates complications in the expert selection process. Since the DAC, to some extent, can greatly affect the final result, the applicants tend to use a single but familiar field rather than multiple fields in order to reduce the risk of being assigned to irrelevant experts outside their field [57]. The same situation occurs in the Big Data field and makes it very difficult to directly measure interdisciplinary attributes without sufficient information or indicators for cross-disciplinary engagement. We conducted a few personal interviews with the officials who worked in the NSFC, and were told that even though NSFC sensed that Big Data should be encouraged to be carried on though interdisciplinary or multidisciplinary research, currently, the Big Data projects granted by NSFC still tended to be more specialized in few particular disciplines, rather than being denoted as interdisciplinary or multidisciplinary studies.

Unlike NSFC, NSF provides one or more program tags to categorize the proposal. Additionally, NSF uses Program Element Codes (PEC) and Program Reference Codes (PRC) to track which NSF programs funded specific awards [58]. This approach to classifying proposals better encompasses the “messiness” of scientific inquiry and may provide a better unit of assessment for measuring interdisciplinary interaction and integration [17]. In Table 7, we find that 77.42% of proposals only offer one main PEC, which shows that most of the Big Data research belongs to a single research area, albeit the other 22.58% are associated with two or more programs. In addition, 65.04% of the proposals are associated with more than one PRC.

**Table 7:** Summary of Program Distribution of Big Data Projects in NSF.

Number of PECs or PRCs	Number of Awards with that # of PECs	% of NSF Awards	Number of Awards with that # of PRCs	% of NSF Awards
0	0	0.00	119	12.92
1	713	77.42	203	22.04
2	145	15.74	300	32.57
3	44	4.78	183	19.87
4	11	1.19	65	7.06
5	6	0.65	27	2.93
6	0	0.00	14	1.52
7	1	0.11	2	0.22
8	1	0.11	2	0.22
9	0	0.00	4	0.43
10	0	0.00	1	0.11
13	0	0.00	1	0.11

In order to measure the degree of interdisciplinarity for a single granted project, we introduce the S-IDR indicator to calculate the degree of interdisciplinarity based on these codes. Borrowing the main ideas from our previous work to calculate the specialization score that encompassed distinct components (Variety, Balance and Disparity), we built an improved diversity indicator inspired by the Hill-type indicator proposed by Zhang et. al. [15] that gave more weight to variety. Higher S-IDR scores represent less interdisciplinarity. The equation of S-IDR is:

$$S-IDR=\frac{1}{1-S}$$ (2)

$$S=\frac{{\sum^{\;}}^{\;}(f_{i}\times f_{j}\times COS(PEC_{i}-PEC_{j}))}{{\sum^{\;}}^{\;}\left(f_{i}\times f_{j}\right)}$$ (3)

In eqs (2) and (3), *i* and *j* represent the number of PECs, *f*_*i*_ and *f*_*j*_ are frequencies of *PEC*_*i*_ and *PEC*_*j*_, *COS (PEC*_*i*_*–PEC*_*j*_*)* is the cosine measure of association between the two PECs to measure the similarity between two PECs, based on a co-occurrence frequency of pairs of PECs in historical NSF proposals. That is, if two PECs frequent appear together in the proposals, then the cosine measure will be relatively high.

**Fig 8:**
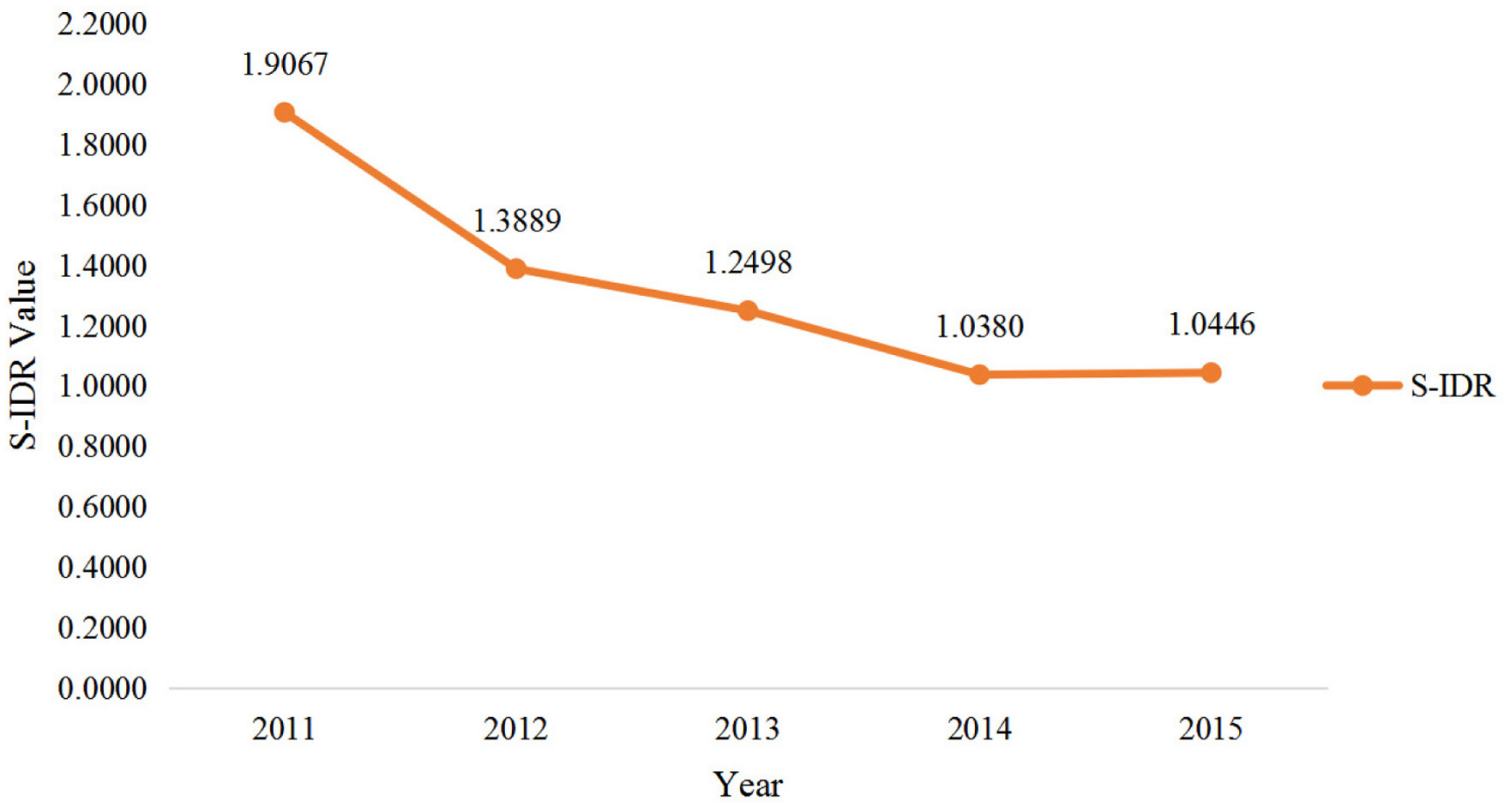
S-IDR Diversity Portfolio during the Period of 2011–2015.

This method of measuring IDR offers a possible way to capture the extent or magnitude of IDR in a given funding portfolio. To determine the interdisciplinarity degree for a certain year or research area, we take the average S-IDR values for the total number of projects belonging to corresponding year or research area. The average S-IDR in the past few years is shown in Fig 8. The figure indicates that the degree of interdisciplinarity has increased from 2011 to 2015. In 2011, the S-IDR value peaked, possibly because investigators localized their studies in the confined scope. During the period of 2014–2015, the curve showed the trend was becoming calm and steady, since there was a time lag between the issued date and the publishing date of proposals.

## Conclusions and Discussion

This paper sought to understand how national scientific funding agencies support rapidly emerging interdisciplinary research. We focused on Big Data, a field that has extended out of the information technology area and grown at a remarkable pace from 2012 to 2015. We applied tech mining, interdisciplinarity evaluation, and network analysis techniques to retrieve and analyze information from funding proposals. In addition, we used a comparative perspective by contrasting the approach of NSF and NSFC to understand how these organizations have made adjustments in response to the rise of the Big Data area.

The proposal activity analysis concluded that both the number and the funding amount of awards are increasing in the US and China. The principal investigators in most awards come from academic organizations, but corporations and individuals from the US have a greater likelihood of being funded by NSF, while research institutes, such as the Chinese Academy of Science, are more apt to be funded by NSFC.

The proposal concentration analysis suggested that Information Science is the core area for Big Data in both the US and China. Engineering and Mathematical & Physical Sciences are two other important disciplines involved with Big Data in the US, while in China, Management Sciences and Earth Sciences are the two other most common disciplines. From a keyword-standpoint perspective, scholars from both the US and China share common research interests in certain Big Data techniques, e.g. “algorithm,” “modeling,” and “machine learning.” However, US proposal titles are more oriented toward engineering and specialized applications while China emphasizes theory and management concepts.

Our results provide some evidences of the notion that different funding agencies use different approaches to ramp-up funding for emerging technologies. Big Data projects granted by NSFC are specialized in particular disciplines rather than being tagged as interdisciplinary or multidisciplinary, but for NSF, the degree of interdisciplinarity is more pronounced. The ability to leverage funding in multiple areas is reflected in NSF’s approach, while NSFC’s method indicates more direct efforts to target resources.

The main contributions of this paper are two-fold. On the one hand, we explored the interdisciplinary issue by using funding data (research proposals) rather than publication or patent data, and presented information relating to engagement of academic individuals, networks, and the evolutionary process of science. We also addressed two different national funding agencies and analyzed two funding datasets to perform this comparison. Such contrasting research methods add a layer of understanding to the diversity of approaches that countries can apply to address rapidly emerging ST&I areas.

In this paper, language distinctions and funding system differences complicated the possibility of a detailed, systematic comparison, and the lack of detailed funding information from NSFC served as a barrier to further comparative analysis with NSF. This imbalanced information should be kept in mind when pursuing such approaches. Building on the logic and methods in this paper, future research could pursue the question of how to further assess the relationships between research funding and research output, and, considerably more challenging, track the interactions between funding and innovation.

## Supporting Information

S1 Table The 87 Research Areas and Their Corresponding Codes of NSFC. (DOCX)

S1 File Raw Data of Big Data Proposals Funded by NSF and NSFC. (XLSX)
