# Supplementary material for: How Does National Scientific Funding Support Emerging Interdisciplinary Research: A Comparison Study of Big Data Research in the US and China
Source: PLoS One. 2016 May 24;11(5):e0154509. doi: 10.1371/journal.pone.0154509 (PMC4878788; doi:10.1371/journal.pone.0154509)
Supplement: S1 Table — (DOCX) [file pone.0154509.s001.docx]

**S1 Table. The 87 Research Areas and Their Corresponding Codes of NSFC**

| **DAC (1st-Level)** | **Research Areas** | **DAC (1st-Level)** | **Research Areas** |
| --- | --- | --- | --- |
| A01 | Mathematics | E06 | Engineering Thermophysics and Energy Utilization |
| A02 | Mechanics | E07 | Electrical Science and Engineering |
| A03 | Astronomy | E08 | Built Environment and Structural Engineering |
| A04 | Physics I | E09 | Water Conservancy Science and Ocean Engineering |
| A05 | Physics II | F01 | Electronics and Information Systems |
| B01 | Inorganic Chemistry | F02 | Computer Science |
| B02 | Organic Chemistry | F03 | Automation |
| B03 | Physical Chemistry | F04 | Semiconductor Science and Information Device |
| B04 | Polymer Science | F05 | Optics and Optoelectronics |
| B05 | Analytical Chemistry | G01 | Management Science and Engineering |
| B06 | Chemical Engineering and Industrial Chemistry | G02 | Business Administration Microorganisms and Infection |
| B07 | Environmental Chemistry | G03 | Macroeconomic Management and Policy |
| C01 | Microbiology | H01 | Respiratory System |
| C02 | Botany | H02 | Circulatory System |
| C03 | Ecology | H03 | Digestive System |
| C04 | Zoology | H04 | Reproductive System/ Perinatology/Newborn |
| C05 | Biophysics, Biochemistry and Molecular Biology | H05 | Urinary System |
| C06 | Genetics and Bioinformatics | H06 | Motor System |
| C07 | Cryobiology | H07 | Endocrine System/Metabolic and Nutritional Support |
| C08 | Immunology | H08 | Hematologic System |
| C09 | Neuroscience | H09 | Nervous System and Mental Illness |
| C10 | Biomechanics and Tissue Engineering | H10 | Medical Immunology |
| C11 | Physiology and Integrative Biology | H11 | Skin and Its appendages |
| C12 | Reproductive Biology and Developmental Biology | H12 | Ophthalmology |
| C13 | Agronomy Foundation and Crop Science | H13 | Otolaryngology Head and Neck Sciences |
| C14 | Plant Protection | H14 | Oral Craniofacial Sciences |
| C15 | Horticulture and Plant Nutrition | H15 | Severe Medical/Trauma/ Burns/Plastic Surgery |
| C16 | Forestry | H16 | Oncology |
| C17 | Animal Husbandry and Grassland Science | H17 | Rehabilitation Medicine |
| C18 | Veterinary Science | H18 | Medical Imaging and Biomedical Engineering |
| C19 | Fisheries Science | H19 | Medical Pathogenic Microorganisms and Infection |
| C20 | Food Science | H20 | Laboratory Medicine |
| C21 | Psychology | H21 | Special Medicine |
| D01 | Geography | H22 | Radiation Medicine |
| D02 | Geology | H23 | Forensic Medicine |
| D03 | Geochemistry | H24 | Endemiology and Occupational Medicine |
| D04 | Geophysics and Space Physics | H25 | Geriatrics |
| D05 | Atmospheric Science | H26 | Preventive Medicine |
| D06 | Marine Science | H27 | Traditional Chinese Medicine |
| E01 | Metallic Materials | H28 | Science of Chinese Pharmacology |
| E02 | Inorganic Non-Metallic Materials | H29 | Chinese and Western Integrative Medicine |
| E03 | Organic Polymer Materials | H30 | Materia Medica |
| E04 | Metallurgy and Mining | H31 | Pharmacology |
| E05 | Mechanical Engineering |  |  |
